# How much does the unguarded X contribute to sex differences in life span?

**DOI:** 10.1002/evl3.292

**Published:** 2022-07-05

**Authors:** Tim Connallon, Isobel J. Beasley, Yasmine McDonough, Filip Ruzicka

**Affiliations:** ^1^ School of Biological Sciences Monash University Clayton VIC 3800 Australia; ^2^ School of BioSciences The University of Melbourne Parkville VIC 3010 Australia; ^3^ Melbourne Integrative Genomics The University of Melbourne Parkville VIC 3010 Australia; ^4^ St. Vincent's Institute of Medical Research Fitzroy VIC 3065 Australia

**Keywords:** Deleterious mutations, evolution of life span, evolutionary theory, inbreeding depression, population genetics, sex chromosomes, sex ratio, sexual dimorphism

## Abstract

Females and males often have markedly different mortality rates and life spans, but it is unclear why these forms of sexual dimorphism evolve. The unguarded X hypothesis contends that dimorphic life spans arise from sex differences in X or Z chromosome copy number (i.e., one copy in the “heterogametic” sex; two copies in the “homogametic” sex), which leads to a disproportionate expression of deleterious mutations by the heterogametic sex (e.g., mammalian males; avian females). Although data on adult sex ratios and sex‐specific longevity are consistent with predictions of the unguarded X hypothesis, direct experimental evidence remains scant, and alternative explanations are difficult to rule out. Using a simple population genetic model, we show that the unguarded X effect on sex differential mortality is a function of several reasonably well‐studied evolutionary parameters, including the proportion of the genome that is sex linked, the genomic deleterious mutation rate, the mean dominance of deleterious mutations, the relative rates of mutation and strengths of selection in each sex, and the average effect of mutations on survival and longevity relative to their effects on fitness. We review published estimates of these parameters, parameterize our model with them, and show that unguarded X effects are too small to explain observed sex differences in life span across species. For example, sex differences in mean life span are known to often exceed 20% (e.g., in mammals), whereas our parameterized models predict unguarded X effects of a few percent (e.g., 1–3% in *Drosophila* and mammals). Indeed, these predicted unguarded X effects fall below statistical thresholds of detectability in most experiments, potentially explaining why direct tests of the hypothesis have generated little support for it. Our results suggest that evolution of sexually dimorphic life spans is predominantly attributable to other mechanisms, potentially including “toxic Y” effects and sexual dimorphism for optimal investment in survival versus reproduction.

## Impact Summary

Females and males are dimorphic for a wide range of traits, including the average lengths of their life spans. Sex differences in life span are both conspicuous and variable among species. For example, in mammals, females live ∼20% longer than males (on average), whereas in birds, males live ∼10% longer than females. One leading explanation for these patterns—the unguarded X hypothesis—argues that sex differences in life span emerge from the distinct sex chromosomes that females and males inherit. For many species, one sex (e.g., female mammals; male birds) carries two copies of each X‐linked gene, whereas the other carries one. Because harmful mutations are partially recessive, the sex with only one copy of the X is more prone to expressing them, and that sex should therefore have a shorter average life span. This prediction of the unguarded X hypothesis is qualitatively consistent with observations of sex‐ratio bias in adults and sexual dimorphism for longevity (e.g., mammalian males have one copy of the X and have shorter lives than females). However, there are other possible explanations for these patterns, making it unclear how much the unguarded X explains species diversity for sex‐specific longevity. We developed a mathematical model for the contribution of unguarded X effects to sex differences in survival and life span, and used data on mutation rates and their effects on survival and fitness to quantify the importance of the unguarded X across species. The model, when combined with current data, suggests that the unguarded X hypothesis cannot explain the conspicuous sex differences in life span that are commonly reported in animal species, particularly vertebrates. Our results suggest that the unguarded X is an unlikely general explanation for the evolution of sexually dimorphic life spans, which gives weight to alternative mechanisms, including “toxic Y” effects and sex differential selection via trade‐offs between survival and reproduction.

Sex differences in life span are widely observed in nature, yet the evolutionary causes of these differences are unresolved and subject to much debate (Maklakov and Lummaa 2013; Bronikowski et al. 2022). Sexually dimorphic mortality rates might have evolved because they are adaptive and reflect sex differences in selection for traits promoting survival versus reproduction (Bonduriansky et al. 2008). Alternatively, sex differences in survival may emerge from sex differences in genetic inheritance (Maklakov and Lummaa 2013). For example, maternal inheritance of mitochondrial genes can lead to the accumulation of mutations that preferentially decrease male life span (i.e., the “mother's curse” hypothesis; Frank and Hurst 1996; Gemmell et al. 2004). Or, because sex chromosomes are asymmetrically inherited between the sexes, the deleterious mutations they harbor can differentially affect female and male fitness components, including survival (i.e., “toxic Y” and “unguarded X” hypotheses; Trivers 1985; Marais et al. 2018; Brown et al. 2020). These scenarios are not mutually exclusive and may all play some role in generating sex differences in life span.

The unguarded X hypothesis, which posits that sexual dimorphism in life span is a simple consequence of the different number of X (or Z) chromosome copies that each sex inherits, is built upon three well‐established findings from evolutionary genetics: (1) random mutations are far more likely to be harmful than beneficial; (2) these deleterious mutations are maintained at low frequencies, at an evolutionary balance between recurrent mutation and purifying selection; and (3) fitness costs of deleterious mutations tend to be partially recessive (Lynch et al. 1999; Charlesworth 2015). These conditions render haploid and inbred individuals more susceptible to expressing harmful genetic variation than outbred and diploid individuals from the same population. Consequently, the sex that is haploid for X‐ or Z‐linked genes (the “heterogametic” sex) should be more susceptible to expressing mutations that decrease survival (all else being equal) than the sex that is diploid for the X or Z chromosome (the “homogametic” sex). Unguarded X effects should, therefore, preferentially reduce male life spans in species with X chromosomes and female life spans in species with Z chromosomes.

The unguarded X hypothesis is consistent with several recently reported empirical patterns. Suggestive evidence for the hypothesis comes from field surveys of adult sex ratios (which may reflect sex differential mortality; Pipoly et al. 2015) and estimates of sex‐specific aging and longevity (Xirocostas et al. 2020; Cayuela et al. 2022). Estimates of adult sex ratios are typically skewed toward the homogametic sex (i.e., females in species with X chromosomes; males in species with a Z; Pipoly et al. 2015; Marais and Lemaitre 2022), as predicted by the unguarded X hypothesis. Similarly, estimates of sex‐specific aging and longevity indicate that the heterogametic sex has a lower mean life span than the homogametic sex (Xirocostas et al. 2020; Marais and Lemaitre 2022). These sex ratio and longevity data span a relatively small fraction of species, with terrestrial vertebrates overrepresented; further study will be required to evaluate whether the reported associations between sex chromosome system and metrics of sex‐specific survival generalize across the tree of life.

Although the patterns summarized above are broadly consistent with the unguarded X hypothesis, it is unclear whether X‐ or Z‐linkage is causal (as noted by Xirocostas et al. 2020). Indeed, taxonomic surveys (as above) can test for correlations between sex chromosome systems and sex‐specific survival, but cannot exclude other plausible contributors. For example, a strong Y‐ or W‐linked genetic basis of mortality, as suggested by recent *Drosophila* experiments (Brown et al. 2020; Nguyen and Bachtrog 2021), could generate taxonomic patterns that parallel those predicted by the unguarded X (Sultanova et al. 2020). Moreover, the sex determination system of a species can potentially affect the evolution of traits that are pertinent to mortality, which may lead to co‐evolved associations between the sex chromosome system and sex‐specific patterns of survival and longevity. Species with X versus Z chromosomes may, for example, be differentially prone to evolving traits that increase male mating success and decrease male survival (Reeve and Pfennig 2003; Albert and Otto 2005).

Because a range of environmental or evolutionary variables may systematically differ between species carrying an X versus a Z chromosome, direct experimental tests for unguarded X effects are essential for evaluating the hypothesis. One experimentally testable prediction of the unguarded X is that inbreeding depression should have a weaker effect on the heterogametic sex (which cannot be inbred for the X or Z) compared to the homogametic sex (which can). Although Vega‐Trejo et al. (2022) recently showed (using meta‐analysis) that inbreeding depression is generally higher in females than males (also see Carazo et al. 2016; Sultanova et al. 2018), this pattern is independent of the sex chromosome system. Moreover, as the authors note, there are other reasons aside from sex linkage why inbreeding depression may differentially affect the sexes, including intrinsic sex differences in the effects of mutations on fitness components (e.g., Whitlock and Agrawal 2009). Direct experimental tests of the unguarded X hypothesis—carried out in *Drosophila* where manipulative experiments of the effect of X‐linked homozygosity on fitness components are feasible—have likewise failed to identify even marginal unguarded X effects (see Eanes et al. 1985; Brengdahl et al. 2018; Narayan et al. 2022).

Here, we argue that there is a more fundamental reason to doubt that unguarded X effects contribute substantially to sexually dimorphic life spans. Building upon prior theory (e.g., Werren 1993; Charlesworth and Charlesworth 1999; Pipoly et al. 2015), we present a simple model that shows that the magnitude of the unguarded X effect depends on several evolutionary parameters that previous studies have estimated, including the genomic deleterious mutation rate, the fraction of the haploid genome that is X‐ or Z‐linked, the average dominance of deleterious mutations, sex biases in mutation and selection, and the average effects of mutations on individual fitness components (e.g., survival and longevity) relative to their effects on total fitness. All of these parameters have been estimated in *Drosophila*, where we predict that unguarded X effects generate sex differences in life span on the order of 3% or less. Furthermore, available data on sex chromosome sizes, mutation rates, and fitness effects, which we review, imply that unguarded X effects in other taxa should generally be of similar magnitude to those predicted for *Drosophila*. The magnitude of our estimates is too small to explain the large sex differences in life span that are often documented in animals (e.g., mammals, where females live on average ∼20% longer than males; Lemaître et al 2020). Hence, although unguarded X effects *should* contribute to sex differences in life span, other factors are likely to be more important in explaining conspicuous patterns of sexual dimorphism for aspects of survival.

## A Model of the Unguarded X

We consider a simple model for the evolution of unguarded X effects arising from a large number of loci maintained at mutation‐selection balance. The focus of the unguarded X hypothesis is on fitness components relevant to mortality in each sex, including survival to reproductive maturity and longevity, and we therefore focus on these fitness components in our model. We present explicit results for the case of male heterogamety assuming that the Y chromosome is degenerate (i.e., XX/XY or XX/XO sex chromosome systems, where males are haploid for X‐linked genes) and the X chromosome is functionally diploid in females (e.g., in mammals with random X inactivation; Deng et al. 2014). Our results are easily reframed to cases of female heterogamety (Z chromosome systems with a degenerate W), by simply reversing the sex labels in our models.

Following previous theory (e.g., Haldane 1937; Connallon 2010), and assuming strong selection relative to mutation ($2\mu_{f,i}+\mu_{m,i}\ll 2s_{f,i}h_{i}+s_{m,i}$, where $\mu_{f,i}$ and $\mu_{m,i}$ represent the female and male mutation rates for the *i*th locus, *s_f,i_* and *s_m,i_* are the selection coefficients, and *h_i_* is the dominance coefficient; Table 1), the equilibrium frequency of a deleterious *a* allele at the *i*th X‐linked locus is

(1) $${\hat{p}}_{X,i}\approx \frac{2\mu_{f,i}+\mu_{m,i}}{2s_{f,i}h_{i}+s_{m,i}}.$$

**Table 1:** Effect of the *i*th X‐linked locus on sex‐specific survival and overall fitness

	X‐Linked Genotype		
	*AA, A*	*Aa*, –	*aa, a*
Female fitness	1	1 – *h_i_s_f,i_*	1 – *s_f,i_*
Male fitness	1	–	1 – *s_m,i_*
Female survival	1	1 – *h_i_s_f,i_α_f,i_*	1 – *s_f,i_α_f,i_*
Male survival	1	–	1 – *s_m,i_α_m,i_*

Parameters include female and male selection coefficients (*s_f,i_*, *s_m,i_*), the dominance coefficient (*h_i_*), and the effect of the mutation on survival relative to its effect on overall fitness (*α_f,i_*, *α_m,i_*, where, e.g., *α_f,i_* = 1 under pure viability selection and *α_f,i_* = 0 under pure fecundity selection).

The contribution of the locus to mean survival or longevity of females and males, respectively, will be ${\bar{w}}_{f,i}\approx 1-2h_{i}s_{f,i}\alpha_{f,i}{\hat{p}}_{X,i}$ and ${\bar{w}}_{m,i}\approx 1-s_{m,i}\alpha_{m,i}{\hat{p}}_{X,i}$, where $\alpha_{j,i}$ represents the effect of the deleterious mutation on the fitness component of the *j*th sex relative to its effect on overall fitness (see Charlesworth 2015; Table 1).

Following Charlesworth and Charlesworth (1999), we assume that loci have multiplicative effects on fitness and fitness components. With *n_X_* X‐linked loci at mutation‐selection balance, the mean value of the female fitness component with respect to the X‐linked loci will be

(2) $${\bar{W}}_{f,X}\approx \exp\left(-\sum_{i=1}^{n_{X}}2h_{i}s_{f,i}\alpha_{f,i}\frac{2\mu_{f,i}+\mu_{m,i}}{2h_{i}s_{f,i}+s_{m,i}}\right),$$

and the mean value of the male fitness component will be

(3) $${\bar{W}}_{m,X}\approx \exp\left(-\sum_{i=1}^{n_{X}}s_{m,i}\alpha_{m,i}\frac{2\mu_{f,i}+\mu_{m,i}}{2h_{i}s_{f,i}+s_{m,i}}\right).$$

The unguarded X effect can be quantified by taking the ratio of equations (2) and (3):

(4) $$\frac{{\bar{W}}_{f,X}}{{\bar{W}}_{m,X}}\approx \exp\left(\sum_{i=1}^{n_{X}}\left(s_{m,i}\alpha_{m,i}-2h_{i}s_{f,i}\alpha_{f,i}\right)\frac{2\mu_{f,i}+\mu_{m,i}}{2h_{i}s_{f,i}+s_{m,i}}\right).$$

Unguarded X effects contribute to lower survival or longevity in the heterogametic sex when equation (4) exceeds unity (${\bar{W}}_{f,X}/{\bar{W}}_{m,X}>1$), which requires that the summation is positive.

### BASELINE PREDICTIONS FOR THE UNGUARDED X

If we assume that the rates and effects of mutations are equal between the sexes (i.e., *s_f,i_* = *s_m,i_* and *α_f,i_* = *α_m,i_*), and that *h_i_*, $\mu_{j,i}$, and $\alpha_{i}$ are independently distributed across X‐linked loci, then equation (4) simplifies to

(5) $$\frac{{\bar{W}}_{f,X}}{{\bar{W}}_{m,X}}\approx \exp\left(3U_{X}\bar{\alpha }\left(\frac{1-2\bar{h}}{1+2\bar{h}}+\frac{8\mathrm{var}\left(h\right)}{{\left(1+2\bar{h}\right)}^{3}}\right)\right),$$

which is derived using a Taylor series expansion, to second order in $h_{i}-\bar{h}$, where $\bar{h}$ is the mean and var(*h*) is the variance of the dominance coefficients of deleterious mutations; $\bar{\alpha }$ is the average effect of mutations on the fitness component relative to overall fitness; and $U_{X}=\frac{1}{3}\;n_{X}E\left[2\mu_{f,i}+\mu_{m,i}\right]$ is the total deleterious mutation rate per X chromosome.

If we further assume that deleterious mutations are generally partially recessive (0 < *h_i_* < 0.5), which is consistent with mutation‐accumulation data (Manna et al. 2011; Charlesworth 2015), then terms of var(*h*) will be small enough to neglect (see the Supporting Information), and equation (5) simplifies to

(6) $$\frac{{\bar{W}}_{f,X}}{{\bar{W}}_{m,X}}\approx \exp\left(\frac{3U_{X}\bar{\alpha }\left(1-2\bar{h}\right)}{1+2\bar{h}}\right).$$

Thus, the magnitude of the unguarded X effect should increase with the X‐linked deleterious mutation rate (*U_X_*) and the mean effect of mutations on the fitness component relative to their effects overall fitness ($\bar{\alpha }$). The unguarded X effect should decrease with mean dominance of deleterious mutations ($\bar{h}$). For the extreme case in which mutations are completely recessive (*h_i_* = 0), and purifying selection is entirely based on mutational effects on viability ($\bar{\alpha }=1$, corresponding to the unlikely scenario where mutations do not affect reproductive fitness components), equation (6) simplifies to $\exp(3U_{X})$, which represents an upper limit for the unguarded X effect that was previously derived by Pipoly et al. (2015).

### SEX DIFFERENCES IN SELECTION AND THE UNGUARDED X

Our baseline model (eq. 6) should provide reasonable predictions for unguarded X effects provided mutations have similar effects on fitness and the fitness components of each sex. We can think of three plausible ways that this assumption may be violated. First, at least some deleterious mutations have sex‐limited fitness effects (e.g., mutations underlying sterility are typically sex limited; Connallon and Clark 2011). Second, stronger sexual selection on one sex (e.g., males) can give rise to systematic sex differences in the strength of purifying selection against deleterious mutations (Whitlock and Agrawal 2009; Grieshop et al. 2021). Third, several studies argue that a lack of dosage compensation, which pertains to some taxa with strongly heteromorphic sex chromosomes, might dampen the phenotypic effects of mutations in the heterogametic (e.g., Charlesworth et al. 1987; Hitchcock and Gardner 2020; Rayner et al. 2021). As we will show, each of these factors tend, if anything, to dampen unguarded X effects, particularly in male‐heterogametic species.

First, consider the case of sex‐limited selection against deleterious mutations. Because we have no reason to expect sex differences in the number of loci affecting viability, we consider the unrealistic but illuminating case where half of X‐linked loci have female‐limited fitness effects and the other half have male‐limited effects (current data, nevertheless, imply that most mutations decrease fitness of both sexes; e.g., Mallet et al. 2011; Sharp and Agrawal 2013). In this idealized case, equations ((2), (3), (4)) simplify to ${\bar{W}}_{f,X}\approx \exp\left(-\frac{3}{2}U_{X}{\bar{\alpha }}_{f}\right)$, ${\bar{W}}_{m,X}\approx \exp\left(-\frac{3}{2}U_{X}{\bar{\alpha }}_{m}\right)$, and ${\bar{W}}_{f,X}/{\bar{W}}_{m,X}\approx \exp\left(\frac{3}{2}U_{X}\left({\bar{\alpha }}_{m}-{\bar{\alpha }}_{f}\right)\right)$. The latter expression suggests that unguarded X effects will vanish, regardless of the dominance of deleterious mutations, when ${\bar{\alpha }}_{m}={\bar{\alpha }}_{f}\;$. Although unguarded X effects could persist when viability‐related fitness components are more important (relative to total fitness) for males than for females (i.e., ${\bar{W}}_{f,X}/{\bar{W}}_{m,X}>1$ when ${\bar{\alpha }}_{m}>{\bar{\alpha }}_{f}$), stronger sexual selection in males should, if anything, tend to disproportionately *decrease* the relative contribution of viability‐related fitness components to male fitness. If so, we expect a higher X‐linked load for the survival or longevity in females compared to males (i.e., ${\bar{W}}_{f,X}/{\bar{W}}_{m,X}<1$ when ${\bar{\alpha }}_{m}<{\bar{\alpha }}_{f}$), thereby reversing the unguarded X effect.

Second, consider the more plausible case of sex‐differential purifying selection, where mutations similarly affect viability in each sex ($\alpha_{f,i}\;s_{f,i}=\alpha_{m,i}\;s_{m,i}$ in our models), but sexual selection leads to stronger purifying selection in males (*s_m,i_* > *s_f,i_*). Following the same approach that we used to derive equation (6) and assuming, for simplicity, that that the ratios *β* = *s_m,i_*/*s_f,i_* = *α_f,i_*/*α_m,i_* are constant across loci, the unguarded X effect becomes

(7) $$\frac{{\bar{W}}_{f,X}}{{\bar{W}}_{m,X}}\approx \exp\left(\frac{3U_{X}{\bar{\alpha }}_{f}\left(1-2\bar{h}\right)}{\beta +2\bar{h}}\right),$$

which, by definition, must have a smaller magnitude than our baseline model (eq. 6) whenever purifying selection is stronger in males (i.e., *β* > 1). To the extent that sexual selection increases *β*, unguarded X effects on survival and longevity should be dampened. Overall, we expect sexual selection to decrease the magnitude of the unguarded X effect in species with XX/XY (or XX/XO) sex chromosome systems. However, the reverse may be true in species with Z chromosomes, in which loci with sex‐limited mutational effects and stronger purifying selection in males than females may accentuate “unguarded Z” effects relative to our baseline model (eq. 6).

Third, consider a scenario in which a lack of dosage compensation systematically reduces the hemizygous fitness effects of X‐linked mutations relative to their effects in homozygous state, as some previous theoretical models have assumed (e.g., Charlesworth et al. 1987; Hitchcock and Gardner 2020). Although mutation accumulation data are needed to validate this assumption, evaluating how it should affect predictions about the direction and magnitude of the unguarded X effect is straightforward (see the Supporting Information, including Fig. S1). Under such conditions, we show that the range of dominance coefficients that will generate an unguarded X effect becomes more restrictive in species lacking dosage compensation relative to those with dosage compensation. Moreover, the magnitude of the unguarded X effect, when it does occur, is dampened in species that lack dosage compensation, except in the unlikely scenario where all deleterious mutations are completely recessive.

## Unguarded X Effects Predicted from Theory and Mutation Data

All of the key parameters in our unguarded X model (*U_X_*, $\bar{h}$, ${\bar{\alpha }}_{f}$, ${\bar{\alpha }}_{m}$, *β*) have been estimated in *Drosophila*, and a subset have also been estimated in other taxa. We first review what we currently know about these parameters (we have greatly benefitted from relatively recent reviews that focus on one or more of these parameters; see Manna et al. 2011; Charlesworth 2015). We subsequently use these estimates to predict the likely contribution of the unguarded X to observed sex differences and life span.

### X‐LINKED AND Z‐LINKED DELETERIOUS MUTATION RATES

The total X‐linked or Z‐linked deleterious mutation rate, *U_X_*, depends on three factors that vary among species: (1) the haploid genomic deleterious rate (*U_H_*), which is most accurately estimated using modern genomic approaches (see below); (2) the proportion of the haploid genome that is X‐linked (*P_X_*) or Z‐linked (*P_Z_*), which has been estimated from karyotype data and annotated, whole‐genome sequences (e.g., Ross et al. 2005; Stiglec et al. 2007; Sultanova et al. 2020); and (3) the ratio of male to female mutation rates ($R_{\mu }={\bar{\mu }}_{m}/{\bar{\mu }}_{f}\;$), which can be estimated from the sequences of parent‐offspring trios or from molecular evolution data (e.g., Ellegren 2007; Hedrick 2007). Taking these factors into account, the total X‐linked deleterious mutation is

(8) $$U_{X}=\frac{2\left(2+R_{\mu }\right)}{3\left(1+R_{\mu }\right)}\;U_{H}P_{X},$$

and the Z‐linked deleterious mutation rate is

(9) $$U_{Z}=\frac{2\left(1+2R_{\mu }\right)}{3\left(1+R_{\mu }\right)}\;U_{H}P_{Z}.$$

In species (like *Drosophila*) where sex biases in mutation are trivial, equations (8) and (9) reduce to $U_{X}=U_{H}\;P_{X}$ and $U_{Z}=U_{H}\;P_{Z}$. In species with strongly male‐biased mutation rates, *U_X_* and *U_Z_* approach the following limits: $\lim_{R_{\mu }\to \infty }U_{X}=\frac{2}{3}\;U_{H}P_{X}$ and $\lim_{R_{\mu }\to \infty }U_{Z}=\frac{4}{3}\;U_{H}P_{Z}$.

Genomic deleterious mutation rate estimates are primarily based on autosomal sequences, where the mutation rates of each sex receive equal weighting. For example, with *n* functional sites on the autosomes, the *haploid genomic deleterious mutation* rate will be $U_{H}=\sum_{i=1}^{n}\frac{1}{2}\left(\mu_{f,i}+\mu_{m,i}\right)=n\bar{\mu }$, where $\bar{\mu }=\frac{1}{2}\left({\bar{\mu }}_{f}+{\bar{\mu }}_{m}\right)$ is the mean mutation rate per nucleotide site (Keightley 2012; Lynch et al. 2016). *U_H_* can be estimated using genome sequence data (Kondrashov and Crow 1993; Haag‐Liautard et al. 2007), in which *n* is based on estimates of genome size and the proportion of sites that are evolutionarily conserved, and $\bar{\mu }$ is estimated by sequencing mutation‐accumulation lines or pedigrees. Current estimates of *U_H_* range from ∼0.5 in *Caenorhabditis elegans*, *Drosophila*, and mice (Denver et al. 2004; Haag‐Liautard et al. 2007; Uchimura et al. 2015) to *U_H_* ∼ 1.1 in humans (Keightley 2012; Dukler et al. 2021). Recent work suggests that *U_H_* is likely to be higher in species with relatively small historical effective population sizes (Lynch et al. 2016; e.g., humans and many other terrestrial vertebrates), and the human estimates may thus be tentatively regarded as near the upper end of *U_H_* among species. Nevertheless, it is currently difficult to generalize about the distribution of *U_H_* among species, and further empirical attention to the issue is warranted.

**Figure 1:**
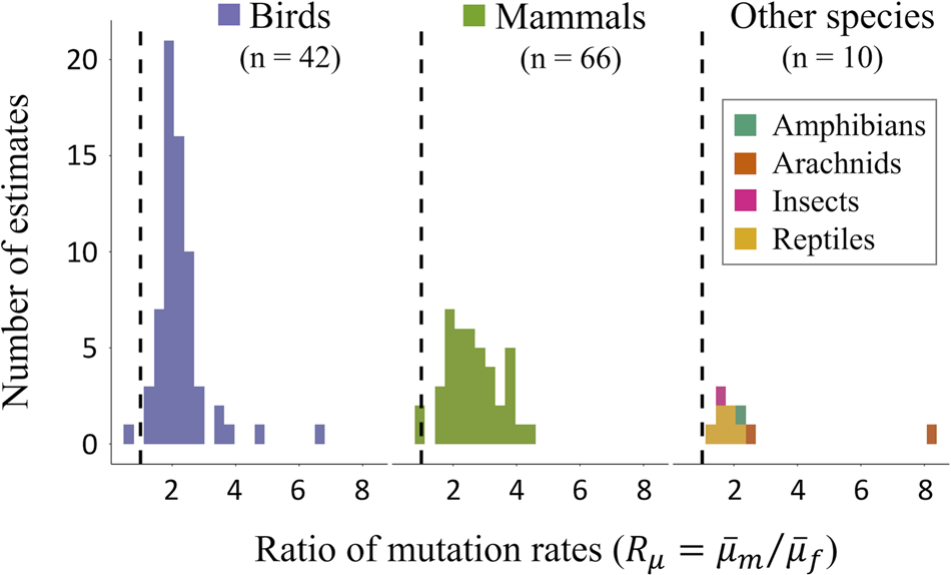
Curated published estimates of species‐specific sex‐biased mutation rates, expressed as the ratio of male to female rates ($R_{\mu }={\bar{\mu }}_{m}/{\bar{\mu }}_{f}$). Results were compiled by surveying the primary literature, including preprints (for details, see the main text and the Supporting Information). We focused on the subset of estimates in which sex‐biased mutation rates could be calculated for individual species or species lineages rather than groups of related species. In cases where there were multiple estimates for a species, we took the average (we excluded a single outlier of $R_{\mu }∼20$ from humans; Wilson Sayres et al. 2011). This process yielded a total of 118 species‐specific estimates (numbers per species group are shown in the figure) from 29 studies (17 mammal, six bird, six other). The vertical broken lines denote equal rates of mutation between the sexes ($R_{\mu }=1$); values exceeding one are male biased $\left(R_{\mu }>1;\;{\bar{\mu }}_{m}>{\bar{\mu }}_{f}\right)$.

Most species fall somewhere along a spectrum of modest‐to‐strong male‐biased mutation rates ($R_{\mu }>1$; Fig. 1), in which $\frac{2}{3}U_{H}P_{X}<U_{X}<U_{H}P_{X}$ and $U_{H}P_{Z}<U_{Z}<\frac{4}{3}U_{H}P_{Z}$, so that male‐biased mutation should somewhat dampen X‐linked mutation rates and elevate Z‐linked rates. We compiled estimates of male‐biased mutation rate from peer‐reviewed articles and preprints up to February 9, 2022, by searching Google Scholar and the Web of Science using key phrases: “sex‐biased mutation,” “male‐biased mutation,” “mutation rate,” “substitution rate,” and “male‐driven evolution.” Where available, we recorded point estimates of $R_{\mu }$, upper and lower bounds for the estimate (e.g., confidence intervals), the species or lineage associated with the estimate, and the method and primary source from which estimates were derived (the entire dataset is provided in the Supporting Information). We excluded studies that were unlikely to be informative for genome‐wide sex biases in mutation (i.e., estimates calculated exclusively using disease‐affecting loci or using organelle genomes) and those whose estimates were later corrected (e.g., Labuda et al. 2010; Lohmueller et al. 2010). Overall, we collected 206 estimates, from 48 studies and 147 species or lineages. The vast majority of estimates were from mammalian (46.3% of all estimates) and avian taxa (44.4%), and were calculated using the molecular evolutionary method (91.2%) proposed by Miyata et al. (1987). The data (Fig. 1) suggest that, typically, $1<R_{\mu }<4$ for mammals and $1<R_{\mu }<3$ in birds. Although fewer estimates are available for other taxa, the full dataset indicated that most species should fall within the $1<R_{\mu }<4$ range, and $1<R_{\mu }<3$ for *Drosophila* species.

### EFFECTS OF DELETERIOUS MUTATIONS ON VIABILITY, LONGEVITY, AND FITNESS

Representative data on the dominance coefficients of deleterious mutations have been obtained from mutation‐accumulation experiments using model organisms (e.g., *Drosophila*, *C. elegans*, and *Saccharomyces cerevisiae*). Although there are many case studies of dominance associated with segregating polymorphisms, these are generally unfit for purpose because they represent a biased sample: such loci have been filtered by natural selection and are undoubtedly enriched for alleles with unusually large (and, thus, empirically discernible) phenotypic effects. Focusing on mutation‐accumulation results, the *Drosophila* data suggest a mean dominance coefficient of $\bar{h}\approx 0.25$ (Charlesworth 2015). A meta‐analysis of estimates spanning four decades, and including data from *Drosophila*, *C. elegans*, and *S. cerevisiae*, yields a similar conclusion ($\bar{h}\approx 0.25$ with a plausible range of $0.18<\bar{h}<0.36$; see Manna et al. 2011). Moreover, these results conform closely to theoretical predictions for the dominance of deleterious mutations arising in populations that have evolved to an optimum (Manna et al. 2011).

Estimates of $\bar{\alpha }$ for several fitness components have been obtained from *Drosophila* mutation‐accumulation experiments (reviewed in table S8 of Charlesworth 2015), including estimates for juvenile viability (i.e., egg‐to‐adult survival, where ${\bar{\alpha }}_{f}\approx {\bar{\alpha }}_{m}\approx 0.3$) and longevity (where $0.1<{\bar{\alpha }}_{f}\approx {\bar{\alpha }}_{m}<0.2$). Estimates of $\bar{\alpha }$ are typically much lower than one‐half, consistent with studies of standing genetic variation that show substantially weaker genotypic effects on viability components relative to total fitness (e.g., Wilton and Sved 1979; Eanes et al. 1985). Although we lack estimates of $\bar{\alpha }$ from vertebrates, the *Drosophila* estimates for viability and longevity may very well exceed those of vertebrate taxa; these longer lived species are likely to have lower rates of preadult mortality relative to *Drosophila* yet high variances in reproductive success among adults, which should cause $\bar{\alpha }$ to be smaller for vertebrate viability.

Finally, sex differences in the overall strength of purifying selection (which affects β in eq. 7) have been estimated in several insect studies, although the most powerful tests have focused on *Drosophila*. Their results suggest that new deleterious mutations tend to harm both sexes, with the strength of purifying selection likely to range from equal between the sexes to roughly 50% stronger in males than females (β∼1.5; Mallet et al. 2011; Sharp and Agrawal 2013; Singh and Agrawal 2022). Recent work further suggests that sex biases in purifying selection are influenced by both the mating system and extrinsic environmental factors that may vary over time, space, or among taxa (Long et al. 2009; Yun et al. 2018).

### PREDICTING THE MAGNITUDE OF UNGUARDED X EFFECTS IN *Drosophila* AND OTHER SPECIES

**Figure 2:**
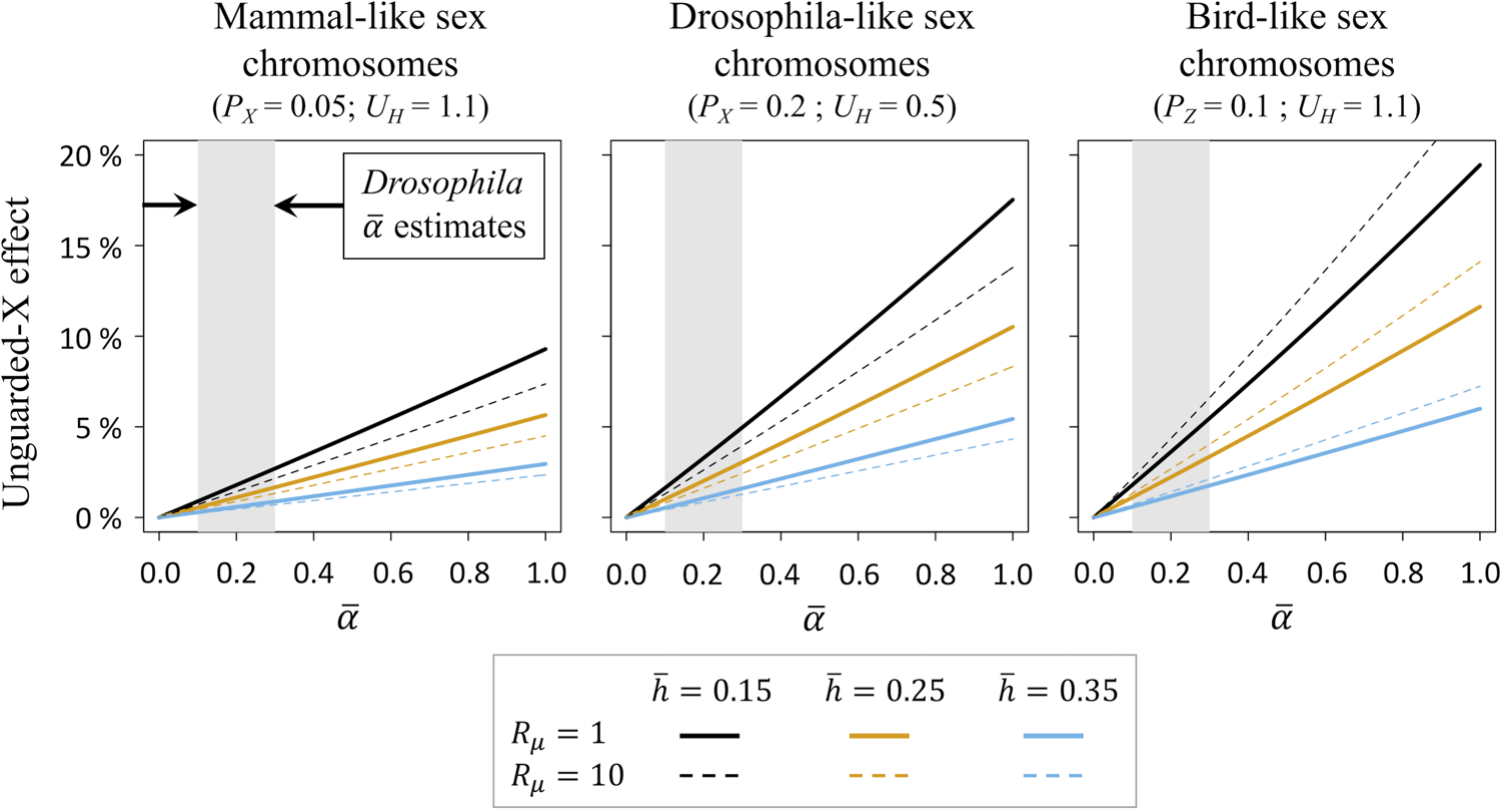
Unguarded X effects in species with mammalian‐like, *Drosophila*‐like, and bird‐like sex chromosomes. Results are shown for plausible values of the haploid genomic deleterious mutation rate (*U_H_* = 1.1 for vertebrates, based on a relatively high estimate from humans: Keightley 2012; Dukler et al. 2021; *U_H_* = 0.5 for *Drosophila*: Haag‐Liautard et al. 2007), with typical sex chromosome sizes for mammals, flies, and birds (bird results are based on the upper end of range of Z chromosome sizes: 0.07 < *P_Z_* < 0.1; Stiglec et al. 2007; Sultanova et al. 2020), and equally strong purifying selection in each sex (*β* = 1). The gray‐shaded regions correspond to the range of $\bar{\alpha }$ estimates obtained from *Drosophila* studies ($0.1<\bar{\alpha }<0.3$; Charlesworth 2015), and the orange lines correspond to dominance values consistent with both theory and mutation accumulation studies from model organisms ($\bar{h}=0.25$; see Manna et al. 2011).

In *Drosophila melanogaster*, the X represents upward of 20% of the genome (*P_X_* ∼ 0.2) and mutation rates are approximately equal between the sexes ($R_{\mu }\;∼\;1$), which (given *U_H_* ∼ 0.5; Haag‐Liautard et al. 2007) suggests an X‐linked deleterious mutation rate of *U_X_* = 0.1. Assuming $\bar{h}\approx 0.25$, and using $\bar{\alpha }=0.3$ (Charlesworth 2015) and *β* = 1 (the approximate lower bound for sex‐specific purifying selection; see above), we estimate that the unguarded X should lead to a 3% elevation in female relative to male viability or life span (${\bar{W}}_{f,X}/{\bar{W}}_{m,X}\approx 1.03$; Fig. 2). Parameterization using lower values of $\bar{\alpha }$ and higher values of *β* decreases the magnitude of the unguarded X effect for *D. melanogaster* (e.g., given $\bar{\alpha }=0.1$ and *β* = 1.5, which are plausible, ${\bar{W}}_{f,X}/{\bar{W}}_{m,X}\approx 1.0075$, corresponding to a sex difference of less than 1%). These small predicted unguarded X effects will become even smaller if a substantial fraction of mutations have sex‐limited effects on life span because sex‐limited loci tend to equalize the contribution of X‐linked mutations to sex differences in mortality (see above).

What about other species? Relative to *D. melanogaster*, the proportion of the genome that is X‐ or Z‐linked is smaller in most other taxa (Tree of Sex Consortium 2014; Sultanova et al. 2020). Because there is little reason to expect major differences in *U_H_*, $\bar{\alpha }$, and $\bar{h}$ between *Drosophila* and other insect species, the smaller sex chromosomes of most insect species (e.g., *P_Z_* ∼ 0.02–0.05 in Lepidoptera: Presgraves 2002; Fraïsse et al. 2017; *P_X_* ∼ 0.05 in fireflies [Coleoptera]: Lower et al. 2017; *P_X_* < 0.08 in cockroaches [Blattodea]: Meisel et al. 2019; *P_X_* < 0.1 is generally implied by the high chromosome numbers of most insects orders: Blackmon et al. 2017; Sylvester et al. 2020) should lead to even smaller unguarded X (or Z) effects than predicted for *D. melanogaster*. Important exceptions include the subset of *Drosophila* species with larger sex chromosomes than *D. melanogaster* (i.e., *P_X_* ∼ 0.4; see Turelli and Begun 1997) and haplo‐diploid species (e.g., Hymenoptera, where *P_X_* = 1).

Although vertebrates also have smaller sex chromosomes than *Drosophila*, they may typically have higher genomic deleterious mutation rates (given the negative relation between *U_H_* and population size, in which the latter is known to be smaller in vertebrates; see Lynch et al. 2016; Buffalo 2021). If we take humans as a representative case for mammals (thus, *U_H_* = 1.1 and *P_X_* = 0.05; Ross et al. 2005; Keightley 2012), and assume that $\bar{h}\approx 0.25$ and $\bar{\alpha }=0.3$, then unguarded X effects are predicted to be even smaller than those of *Drosophila* (i.e., ${\bar{W}}_{f,X}/{\bar{W}}_{m,X}\approx 1.017$ or 1.7% longer life span for females; Fig. 2); male‐biased mutation and purifying selection further reduce the predicted effect.

Among vertebrates, our model predicts that birds should have higher potential for unguarded X effects because their sex chromosomes are somewhat large compared to mammals and their genomic deleterious mutation rates may be comparable to those of mammals (Fig. 2; but note that *U_H_* may be substantially smaller in birds compared to humans; Smeds et al. 2016). Male‐biased mutation rates ($R_{\mu }>1$, as is typical for birds; Fig. 1) should further elevate Z‐linked deleterious mutation rates in avian taxa (i.e., based on eq. 9). Using a relatively high value of *P_Z_* = 0.1 for the Z‐linked proportion of the genome (0.07 < *P_Z_* < 0.1 in birds; Stiglec et al. 2007; Sultanova et al. 2020), a high genomic deleterious mutation rate (*U_H_* = 1.1, which is comparable to humans), strongly male‐biased mutation ($R_{\mu }=4$), and the remaining parameters based on *Drosophila* estimates ($\bar{h}\approx 0.25$, $\bar{\alpha }=0.3$, *β* = 1.5), the unguarded X is predicted to increase male longevity by 5.2% relative to females (recall that females are haploid). The prediction declines to ∼3.3% in the absence of systematic sex differences in mutation or purifying selection, and further declines with lower values for *P_Z_* and *U_H_* (e.g., *P_Z_* = 0.07 and *U_H_* < 1, both of which are plausible).

## Discussion

Large sex differences in survival, ageing, and longevity have been reported in surveys of adult sex ratios and sex‐specific life spans, and these differences often qualitatively agree with the unguarded X hypothesis. However, our population genetic models of the unguarded X effect, along with current parameter estimates for the rates and effects of deleterious mutations, fall considerably short of explaining the relatively large differences in mortality that are observed in nature (Pipoly et al. 2015 reached a similar conclusion concerning adult sex ratios, using a specific case of our model; see eq. 6 and surrounding text). For example, our predictions for mammals (∼1–2% higher mean female survival) fall far below the 20% elevation in mean female life span that has been reported in empirical surveys of mammalian longevity (Lemaître et al 2020; Xirocostas et al. 2020). Predictions for birds (∼3–5% higher male survival) similarly fall short of the average sex difference in longevity (∼10%) based on avian data included in Xirocostas et al. (2020). Predictions for *D. melanogaster* (∼1–3% higher female survival) are insufficiently large to explain the extent of female‐biased longevity in laboratory populations of *D. melanogaster* (i.e., data compiled in Ziehm et al. 2015 suggest that female life span is typically >10% longer than male life span; see the Supporting Information), and may further help explain the negligible estimates of X‐linked inbreeding depression in females with respect to viability and longevity (Eanes et al. 1985; Brengdahl et al. 2018; Narayan et al. 2022). Although these experiments were by no means small, they were underpowered to detect levels of X‐linked inbreeding depression predicted by our model (Eanes et al. 1985 could only reject effects exceeding 5%).

Two additional observations imply that the unguarded X is unlikely to provide a general explanation for sex differences in longevity. First, extensive sex differences for ageing and life span are observed in species with largely homomorphic sex chromosomes, including higher mortality rates in the heterogametic sex (Cayuela et al. 2022), which cannot be explained by unguarded X effects. Second, although birds are predicted (by unguarded X theory) to exhibit stronger sex differences in life span than mammals, data support the opposite pattern (the sex difference in life span in birds is roughly half the average difference observed in mammals; Lemaître et al. 2020; Xirocostas et al. 2020).

Does this mean that the unguarded X hypothesis is dead? Not entirely. Although current theory and data suggest the hypothesis is unlikely to explain broad taxonomic patterns of sex‐specific longevity, several factors might elevate its importance for some species. First, species with exceptionally large X chromosomes, including haplodiploids, could experience moderate or even large unguarded X effects. For example, with mutation parameters similar to those of *Drosophila* (e.g., $\bar{h}=0.25$, $U_{H}=0.5$, $\bar{\alpha }=0.3$, $R_{\mu }=\beta =1$), our model predicts a ∼15% reduction in male relative to female survival in haplodiploid species, with similar reductions in viability expected for inbred females. Although such effect sizes should be statistically discernible in reasonably sized experiments, most tests for inbreeding depression in haplodiploid species suggest that its effect on survival and longevity is typically weak (Saito et al. 2000; Mori et al. 2005; Tien et al. 2015; for an exception, see Henter 2003). Second, synergistic epistasis between deleterious mutations (which our models do not consider) can amplify inbreeding depression and genetic loads expressed by haploid (or heterogametic) individuals relative to diploid individuals, particularly if mutations are strongly recessive ($\bar{h}<0.25$; Kondrashov and Crow 1991). Although it is unclear how widespread synergistic epistasis is (Charlesworth and Charlesworth 1999; de Visser and Elena 2007; Domínguez‐García et al. 2019), epistatic effects on viability could substantially elevate unguarded X effects in species where sex chromosomes are large (and, hence, *U_X_* is also large; Kondrashov and Crow 1991; Charlesworth 1998). Finally, estimates of $\bar{h}$ and especially $\bar{\alpha }$ are primarily derived from *Drosophila* studies (Manna et al. 2011; Charlesworth 2015), and it is unclear how much these parameters vary among species. Although there is no obvious reason for $\bar{h}$ to differ among taxa, there is good reason to expect variation in $\bar{\alpha }$, owing to among‐species variability in the contribution of preadult mortality to total fitness variance. In plants and broadcast spawning marine organisms, juvenile mortality will often be exceptionally high, and in such cases, viability selection may represent the dominant component of overall selection, causing $\bar{\alpha }$ to be larger than the *Drosophila* estimates imply. Those species that also carry heteromorphic sex chromosomes (e.g., *Silene latifolia*: Charlesworth 2016; *Paracentrotus lividus*: Lipani et al. 1996) are potential candidates for substantial unguarded X effects.

Our main conclusion—that the unguarded X hypothesis is unlikely to explain diverse and often conspicuous patterns of sexual dimorphism for life span among species—gives weight to prominent alternative hypotheses for the evolutionary origins of sexually dimorphic survival and longevity. One such alternative is the toxic Y hypothesis, which posits that nonrecombining Y and W chromosomes serve as reservoirs for accumulation of mutations, which (owing to male‐ and female‐limited inheritance of the Y and W, respectively) solely decrease survival of the heterogametic sex. Although this hypothesis is relatively young, it is supported by experimental evidence from *Drosophila* (Brown et al. 2020; Nguyen and Bachtrog 2021), and there is suggestive evidence that it might apply broadly to other taxa (Sultanova et al. 2020; Peona et al. 2021). The toxic Y effect has yet to be formally modeled, which would help identify biological conditions mediating its magnitude and taxa where it is likely to be particularly important. A second major hypothesis is that sexually dimorphic longevity reflects a fundamental sex difference in the optimal rate of investment in survival versus reproduction (Maklakov and Lummaa 2013). Although it is widely thought that such sexually dimorphic optima often favor the evolution of sex‐differential mortality, it remains an open question whether such scenarios are also likely to play out differently in species with distinct sex determination systems. There are plausible theoretical arguments that could explain why the sex chromosome system of a species might influence the evolution of sexual dimorphism for traits correlated with susceptibility to pathogens, predators, and other sources of mortality (Hastings 1994; Andrés and Morrow 2003; Reeve and Pfennig 2003; Kirkpatrick and Hall 2004; Albert and Otto 2005; Brom et al. 2018). Such scenarios should be taken seriously as potential factors contributing to predictable differences in life span between the heterogametic and homogametic sex.

## AUTHOR CONTRIBUTIONS

TC, IB, YM, and FR conceptualized the study. TC developed the model. IB and YM compiled the sex‐biased mutation estimates. TC, IB, YM, and FR reviewed the relevant scientific literature. TC wrote the manuscript with extensive input from IB, YM, and FR.

## CONFLICT OF INTEREST

The authors declare no conflict of interest.

## DATA ARCHIVING

The compiled dataset of sex‐biased mutation estimates is provided in full in the Supporting Information. Data and code used to create Figure 1 can be found in the GitHub repository: https://github.com/IJbeasley/unguarded_x_journal_club.

## Supporting information

Supplementary Information

Supplementary Information

## References

[evl3292-bib-0001] Albert, A.Y.K. & Otto, S.P. (2005) Sexual selection can resolve sex‐linked sexual antagonism. Science, 310, 119–121. 16210543 10.1126/science.1115328

[evl3292-bib-0002] Andrés, J.A. & Morrow, E.H. (2003) The origin of interlocus sexual conflict: is sex‐linkage important?. J. Evol. Biol, 16, 219–223. 14635860 10.1046/j.1420-9101.2003.00525.x

[evl3292-bib-0003] Blackmon, H. , Ross, L. & Bachtrog, D. (2017) Sex determination, sex chromosomes, and karyotype evolution in insects. J. Hered, 108, 78–93. 27543823 10.1093/jhered/esw047 PMC6281344

[evl3292-bib-0004] Bonduriansky, R. , Maklakov, A. , Zajitschek, F. & Brooks, R. (2008) Sexual selection, sexual conflict and the evolution of ageing and life span. Funct. Ecol, 22, 443–453.

[evl3292-bib-0005] Brengdahl, M. , Kimber, C.M. , Maguire‐Baxter, J. & Friberg, U. (2018) Sex differences in life span: females homozygous for the X chromosome do not suffer the shorter life span predicted by the unguarded X hypothesis. Evolution, 72, 568–577. 29430636 10.1111/evo.13434

[evl3292-bib-0006] Brom, T. , Massot, M. & Laloi, D. (2018) The sex chromosome system can influence the evolution of sex‐biased dispersal. J. Evol. Biol, 31, 1377–1385. 29927019 10.1111/jeb.13340

[evl3292-bib-0007] Bronikowski, A.M. , Meisel, R.P. , Biga, P.R. , Walters, J.R. , Mank, J.E. , Larschan, E. , et al. (2022) Sex‐specific aging in animals: perspective and future directions. Aging Cell, 21:e13542. 35072344 10.1111/acel.13542 PMC8844111

[evl3292-bib-0008] Brown, E.J. , Nguyen, A.H. & Bachtrog, D. (2020) The Y chromosome may contribute to sex‐specific ageing in *Drosophila* . Nat. Ecol. Evol, 4, 853–862. 32313175 10.1038/s41559-020-1179-5 PMC7274899

[evl3292-bib-0009] Buffalo, V. (2021) Quantifying the relationship between genetic diversity and population size suggests natural selection cannot explain Lewontin's Paradox. Elife, 10:e67509. 34409937 10.7554/eLife.67509 PMC8486380

[evl3292-bib-0010] Carazo, P. , Green, J. , Sepil, I. , Pizzari, T. & Wigby, S. (2016) Inbreeding removes sex differences in lifespan in a population of *Drosophila melanogaster* . Biol. Lett, 12:20160337. 27354712 10.1098/rsbl.2016.0337 PMC4938057

[evl3292-bib-0011] Cayuela, H. , Lemaître, J.‐F. , Léna, J.‐P. , Ronget, V. , Martínez‐Solano, I. , Muths, E. , et al. (2022) Sex‐related differences in aging rate are associated with sex chromosome system in amphibians. Evolution, 76, 346–356. 34878663 10.1111/evo.14410 PMC9304222

[evl3292-bib-0012] Charlesworth, B. (1998) The effect of synergistic epistasis on the inbreeding load. Genet. Res, 71, 85–89. 9674385 10.1017/s0016672398003140

[evl3292-bib-0013] Charlesworth, B. (2015) Causes of natural variation in fitness: evidence from studies of *Drosophila* populations. Proc. Natl. Acad. Sci. USA, 112, 1662–1669. 25572964 10.1073/pnas.1423275112 PMC4330759

[evl3292-bib-0014] Charlesworth, B. & Charlesworth, D. (1999) The genetic basis of inbreeding depression. Genet. Res, 74, 329–340. 10689809 10.1017/s0016672399004152

[evl3292-bib-0015] Charlesworth, B. , Coyne, J.A. & Barton, N.H. (1987) The relative rates of evolution of sex chromosomes and autosomes. Am. Nat, 130, 113–146.

[evl3292-bib-0016] Charlesworth, D. (2016) Plant sex chromosomes. Annu. Rev. Plant Biol, 67, 397–420. 26653795 10.1146/annurev-arplant-043015-111911

[evl3292-bib-0017] Connallon, T. (2010) Genic capture, sex linkage, and the heritability of fitness. Am. Nat, 175, 564–576. 20331359 10.1086/651590

[evl3292-bib-0018] Connallon, T. & Clark, A.G. (2011) Association between sex‐biased gene expression and mutations with sex‐specific phenotypic consequences in *Drosophila* . Genome. Biol. Evol, 3, 151–155. 21292631 10.1093/gbe/evr004 PMC3048362

[evl3292-bib-0019] Deng, X. , Berletch, J.B. , Nguyen, D.K. & Disteche, C.M. (2014) X chromosome regulation: diverse patterns in development, tissues and disease. Nat. Rev. Genet, 15, 367–378. 24733023 10.1038/nrg3687 PMC4117651

[evl3292-bib-0020] Denver, D.R. , Morris, K. , Lynch, M. & Thomas, W.K. (2004) High mutation rate and predominance of insertions in the *Caenorhabditis elegans* nuclear genome. Nature, 430, 679–682. 15295601 10.1038/nature02697

[evl3292-bib-0021] de Visser, J. & Elena, S.F. (2007) The evolution of sex: empirical insights into the roles of epistasis and drift. Nat. Rev. Genet, 8, 139–149. 17230200 10.1038/nrg1985

[evl3292-bib-0022] Domínguez‐García, S. , García, C. , Quesada, H. & Caballero, A. (2019) Accelerated inbreeding depression suggests synergistic epistasis for deleterious mutations in *Drosophila melanogaster* . Heredity, 123, 709–722. 31477803 10.1038/s41437-019-0263-6 PMC6834575

[evl3292-bib-0023] Dukler, N. , Mughal, M.R. , Ramani, R. , Huang, Y.F. & Siepel, A. (2021) Extreme purifying selection against point mutations in the human genome. bioRxiv, 10.1101/2021.08.23.457339. PMC9314448 35879308

[evl3292-bib-0024] Eanes, W.F. , Hey, J. & Houle, D. (1985) Homozygous and hemizygous viability variation on the X chromosome of *Drosophila melanogaster* . Genetics, 111, 831–844. 17246306 10.1093/genetics/111.4.831 PMC1202675

[evl3292-bib-0025] Ellegren, H. (2007) Characteristics, causes and evolutionary consequences of male‐biased mutation. Proc. R. Soc. B Biol. Sci, 274, 1–10. 10.1098/rspb.2006.3720 PMC1679872 17134994

[evl3292-bib-0026] Fraïsse, C. , Picard, M.A.L. & Vicoso, B. (2017) The deep conservation of the Lepidoptera Z chromosome suggests a non‐canonical origin of the W. Nat. Commun, 8, 1486. 29133797 10.1038/s41467-017-01663-5 PMC5684275

[evl3292-bib-0027] Frank, S.A. & Hurst, L.D. (1996) Mitochondria and male disease. Nature, 383, 224. 8805695 10.1038/383224a0

[evl3292-bib-0028] Gemmell, N.J. , Metcalf, V.J. & Allendorf, F.W. (2004) Mother's curse: the effect of mtDNA on individual fitness and population viability. Trends Ecol. Evol, 19, 238–244. 16701262 10.1016/j.tree.2004.02.002

[evl3292-bib-0029] Grieshop, K. , Maurizio, P.L. , Arnqvist, G. & Berger, D. (2021) Selection in males purges the mutation load on female fitness. Evol. Lett, 5, 328–343 34367659 10.1002/evl3.239 PMC8327962

[evl3292-bib-0030] Haag‐Liautard, C. , Dorris, M. , Maside, X. , Macaskill, S. , Halligan, D.L. , Charlesworth, B. , et al. (2007) Direct estimation of per nucleotide and genomic deleterious mutation rates in *Drosophila* . Nature, 445, 82–85. 17203060 10.1038/nature05388

[evl3292-bib-0031] Haldane, J.B.S. (1937) The effect of variation on fitness. Am. Nat, 71, 337–349.

[evl3292-bib-0032] Hastings, I.M. (1994) Manifestations of sexual selection may depend on the genetic basis of sex determination. Proc. R. Soc. B Biol. Sci, 258, 83–87. 10.1098/rspb.1994.0146 7997460

[evl3292-bib-0033] Hedrick, P.W. (2007) Sex: differences in mutation, recombination, selection, gene flow, and genetic drift. Evolution, 61, 2750–2771. 17976181 10.1111/j.1558-5646.2007.00250.x

[evl3292-bib-0034] Henter, H.J. (2003) Inbreeding depression and haplodiploidy: experimental measures in a parasitoid and comparisons across diploid and haplodiploid insect taxa. Evolution, 57, 1793–1803. 14503621 10.1111/j.0014-3820.2003.tb00587.x

[evl3292-bib-0035] Hitchcock, T.J. & Gardner, A. (2020) A gene's‐eye view of sexual antagonism. Proc. R. Soc. B Biol. Sci, 287:20201633. 10.1098/rspb.2020.1633 PMC7575522 32781951

[evl3292-bib-0036] Keightley, P.D. (2012) Rates and fitness consequences of new mutations in humans. Genetics, 190, 295–304. 22345605 10.1534/genetics.111.134668 PMC3276617

[evl3292-bib-0037] Kirkpatrick, M. & Hall, D.W. (2004) Sexual selection and sex linkage. Evolution, 58, 683–691. 15154544 10.1111/j.0014-3820.2004.tb00401.x

[evl3292-bib-0038] Kondrashov, A.S. & Crow, J.F. (1991) Haploidy or diploidy: which is better? Nature, 351, 314–315. 2034273 10.1038/351314a0

[evl3292-bib-0039] ———. (1993) A molecular approach to estimating the human deleterious mutation rate. Hum. Mutat, 2, 229–234. 8364591 10.1002/humu.1380020312

[evl3292-bib-0040] Labuda, D. , Lefebvre, J.F. , Nadeau, P. & Roy‐Gagnon, M.H. (2010) Female‐to‐male breeding ratio in modern humans‐an analysis based on historical recombinations. Am. J. Hum. Genet, 86, 353–363. 20188344 10.1016/j.ajhg.2010.01.029 PMC2833377

[evl3292-bib-0041] Lemaître, J.F. , Ronget, V. , Tidière, M. , Allainé, D. , Berger, V. , Cohas, A. , et al. (2020) Sex differences in adult lifespan and aging rates of mortality across wild mammals. Proc. Natl. Acad. Sci. USA, 117, 8546–8553 32205429 10.1073/pnas.1911999117 PMC7165438

[evl3292-bib-0042] Lipani, C. , Vitturi, R. , Sconzo, G. & Barbata, G. (1996) Karyotype analysis of the sea urchin *Paracentrotus lividus* (Echinodermata): evidence for a heteromorphic chromosome sex mechanism. Mar. Biol, 127, 67–72.

[evl3292-bib-0043] Lohmueller, K.E. , Degenhardt, J.D. & Keinan, A. (2010) Sex‐averaged recombination and mutation rates on the X chromosome: a comment on Labuda et al. Am. J. Hum. Genet, 86, 978–981. 20541048 10.1016/j.ajhg.2010.03.021 PMC3032063

[evl3292-bib-0044] Long, T.A.F. , Pischedda, A. , Stewart, A.D. & Rice, W.R. (2009) A cost of sexual attractiveness to high‐fitness females. PLoS Biol, 7:e1000254. 19997646 10.1371/journal.pbio.1000254 PMC2780925

[evl3292-bib-0045] Lower, S.S. , Johnston, J.S. , Stanger‐Hall, K.F. , Hjelmen, C.E. , Hanrahan, S.J. , Korunes, K. , et al. (2017) Genome size in north american fireflies: substantial variation likely driven by neutral processes. Genome. Biol. Evol, 9, 1499–1512. 28541478 10.1093/gbe/evx097 PMC5499882

[evl3292-bib-0046] Lynch, M. , Blanchard, J. , Houle, D. , Kibota, T. , Schultz, S. , Vassilieva, L. , et al. (1999) Perspective: spontaneous deleterious mutation. Evolution, 53, 645–663. 28565627 10.1111/j.1558-5646.1999.tb05361.x

[evl3292-bib-0047] Lynch, M. , Ackerman, M.S. , Gout, J.F. , Long, H. , Sung, W. , Thomas, W.K. , et al. (2016) Genetic drift, selection and the evolution of the mutation rate. Nat. Rev. Genet, 17, 704–714. 27739533 10.1038/nrg.2016.104

[evl3292-bib-0048] Maklakov, A.A. & Lummaa, V. (2013) Evolution of sex differences in lifespan and aging: causes and constraints. Bioessays, 35, 717–724. 23733656 10.1002/bies.201300021

[evl3292-bib-0049] Mallet, M.A. , Bouchard, J.M. , Kimber, C.M. & Chippindale, A.K. (2011) Experimental mutation‐accumulation on the X chromosome of *Drosophila melanogaster* reveals stronger selection on males than females. BMC Evol. Biol, 11, 156. 21645375 10.1186/1471-2148-11-156 PMC3134001

[evl3292-bib-0050] Manna, F. , Martin, G. & Lenormand, T. (2011) Fitness landscapes: an alternative theory for the dominance of mutation. Genetics, 189, 923–937. 21890744 10.1534/genetics.111.132944 PMC3213354

[evl3292-bib-0051] Marais, G.A.B. & Lemaître, J.F. (2022) Sex chromosomes, sex ratios and sex gaps in longevity in plants. Philos. Trans. R. Soc. B Biol. Sci, 377:20210219. 10.1098/rstb.2021.0219 PMC8935291 35306888

[evl3292-bib-0052] Marais, G.A.B. , Gaillard, J.M. , Vieira, C. , Plotton, I. , Sanlaville, D. , Gueyffier, F. , et al. (2018) Sex gap in aging and longevity: can sex chromosomes play a role? Biol. Sex Differ, 9, 33. 30016998 10.1186/s13293-018-0181-y PMC6050741

[evl3292-bib-0053] Meisel, R.P. , Delclos, P.J. & Wexler, J.R. (2019) The X chromosome of the German cockroach, *Blattella germanica*, is homologous to a fly X chromosome despite 400 million years divergence. BMC Biol, 17, 100. 31806031 10.1186/s12915-019-0721-x PMC6894488

[evl3292-bib-0054] Miyata, T. , Hayashida, H. , Kuma, K. , Mitsuyasu, K. & Yasunaga, T. (1987) Male‐driven molecular evolution: a model and nucleotide sequence analysis. Cold Spring Harb. Symp. Quant. Biol, 52, 863–867. 3454295 10.1101/sqb.1987.052.01.094

[evl3292-bib-0055] Mori, K. , Saito, Y. , Sakagami, T. & Sahara, K. (2005) Inbreeding depression of female fecundity by genetic factors retained in natural populations of a male‐haploid social mite (Acari: Tetranychidae). Exp. Appl. Acarol, 36, 15–23. 16082920 10.1007/s10493-004-8151-y

[evl3292-bib-0056] Narayan, V.P. , Wilson, A.J. & Chenoweth, S.F. (2022) Genetic and social contributions to sex differences in lifespan in *Drosophila serrata* . J. Evol. Biol, 35, 657–663. 35290690 10.1111/jeb.13992 PMC9314142

[evl3292-bib-0057] Nguyen, A.H. & Bachtrog, D. (2021) Toxic Y chromosome: increased repeat expression and age‐associated heterochromatin loss in male *Drosophila* with a young Y chromosome. PLoS Genet, 17:e1009438. 33886541 10.1371/journal.pgen.1009438 PMC8061872

[evl3292-bib-0058] Peona, V. , Palacios‐Gimenez, O.M. , Blommaert, J. , Liu, J. , Haryoko, T. , Jønsson, K.A. , et al. (2021) The avian W chromosome is a refugium for endogenous retrovirusus with likely effects on female‐biased mutational load and genetic incompatibilities. Philos. Trans. R. Soc. B Biol. Sci, 376:20200186. 10.1098/rstb.2020.0186 PMC8310711 34304594

[evl3292-bib-0059] Pipoly, I. , Bókony, V. , Kirkpatrick, M. , Donald, P.F. , Székely, T. & Liker, A. (2015) The genetic sex‐determination system predicts adult sex ratios in tetrapods. Nature, 527, 91–94. 26444239 10.1038/nature15380

[evl3292-bib-0060] Presgraves, D.C. (2002) Patterns of postzygotic isolation in Lepidoptera. Evolution, 56, 1168–1183. 12144018 10.1111/j.0014-3820.2002.tb01430.x

[evl3292-bib-0061] Rayner, J.G. , Hitchcock, T.J. & Bailey, N.W. (2021) Variable dosage compensation is associated with female consequences of an X‐linked, male‐beneficial mutation. Proc. R. Soc. B Biol. Sci, 288:20210355. 10.1098/rspb.2021.0355 PMC8059673 33757350

[evl3292-bib-0062] Reeve, H.K. & Pfennig, D.W. (2003) Genetic biases for showy males: are some genetic systems especially conducive to sexual selection? Proc. Natl. Acad. Sci. USA, 100, 1089–1094. 12540829 10.1073/pnas.0337427100 PMC298731

[evl3292-bib-0063] Ross, M.T. , Grafham, D.V. , Coffey, A.J. , Scherer, S. , McLay, K. , Muzny, D. , et al. (2005) The DNA sequence of the human X chromosome. Nature, 434, 325–337. 15772651 10.1038/nature03440 PMC2665286

[evl3292-bib-0064] Saito, Y. , Sahara, K. & Mori, K. (2000) Inbreeding depression by recessive deleterious genes affecting female fecundity of a haplo‐diploid mite. J. Evol. Biol, 13, 668–678.

[evl3292-bib-0065] Sharp, N.P. & Agrawal, A.F. (2013) Male‐biased fitness effects of spontaneous mutations in *Drosophila melanogaster* . Evolution, 67, 1189–1195. 23550766 10.1111/j.1558-5646.2012.01834.x

[evl3292-bib-0066] Singh, A. & Agrawal, A.F. (2022) Sex‐specific variance in fitness and the efficacy of selection. Am. Nat, 199, 587–602. 35472021 10.1086/719015

[evl3292-bib-0067] Smeds, L. , Qvarnström, A. & Ellegren, H. (2016) Direct estimate of the rate of germline mutation in a bird. Genome Res, 26, 1211–1218. 27412854 10.1101/gr.204669.116 PMC5052036

[evl3292-bib-0068] Stiglec, R. , Ezaz, T. & Graves, J.A.M. (2007) A new look at the evolution of avian sex chromosomes. Cytogenet. Genome Res, 117, 103–109. 17675850 10.1159/000103170

[evl3292-bib-0069] Sultanova, Z. , Andic, M. & Carazo, P. (2018) The “unguarded‐X” and the genetic architecture of lifespan: inbreeding results in a potentially maladaptive sex‐specific reduction of female lifespan in *Drosophila melanogaster* . Evolution, 27, 540–552. 10.1111/evo.13426 29336481

[evl3292-bib-0070] Sultanova, Z. , Downing, P.A. & Carazo, P. (2020) Genetic sex determination and sex‐specific lifespan in tetrapods—evidence of a toxic Y effect. bioRxiv, 10.1101/2020.03.09.983700. PMC10107984 36537352

[evl3292-bib-0071] Sylvester, T. , Hjelmen, C.E. , Hanrahan, S.J. , Lenhart, P.A. , Johnston, J.S. & Blackmon, H. (2020) Lineage‐specific patterns of chromosome evolution are the rule not the exception in Polyneoptera insects. Proc. R. Soc. B Biol. Sci, 287:20201388. 10.1098/rspb.2020.1388 PMC7542826 32993470

[evl3292-bib-0072] Tien, N.S.H. , Sabelis, M.W. & Egas, M. (2015) Inbreeding depression and purging in a haplodiploid: gender‐related effects. Heredity, 114, 327–332. 25407077 10.1038/hdy.2014.106 PMC4815584

[evl3292-bib-0073] Tree of Sex Consortium . (2014) Tree of Sex: a database of sexual systems. Sci. Data, 1:140015. 25977773 10.1038/sdata.2014.15 PMC4322564

[evl3292-bib-0074] Trivers, R. (1985) Social evolution. The Benjamin/Cummings Publishing Company, Menlo Park, CA.

[evl3292-bib-0075] Turelli, M. & Begun, D.J. (1997) Haldane's rule and X‐chromosome size in *Drosophila* . Genetics, 147, 1799–1815. 9409837 10.1093/genetics/147.4.1799 PMC1208347

[evl3292-bib-0076] Uchimura, A. , Higuchi, M. , Minakuchi, Y. , Ohno, M. , Toyoda, A. , Fujiyama, A. , et al. (2015) Germline mutation rates and the long‐term phenotypic effects of mutation accumulation in wild‐type laboratory mice and mutator mice. Genome Res, 25, 1125–1134. 26129709 10.1101/gr.186148.114 PMC4509997

[evl3292-bib-0077] Vega‐Trejo, R. , de Boer, R.A. , Fitzptrick, J.L. & Kotrschal, A. (2022) Sex‐specific inbreeding depression: a meta‐analysis. Ecol. Lett, 25, 1009–1026. 35064612 10.1111/ele.13961 PMC9304238

[evl3292-bib-0078] Werren, J.H. (1993) The evolution of inbreeding in haplodiploid organisms. Pp. 42–59 *in* Thornhill, N.W. , ed. The natural history of inbreeding and outbreeding: theoretical and empirical perspectives. University of Chicago Press, Chicago.

[evl3292-bib-0079] Whitlock, M.C. & Agrawal, A.F. (2009) Purging the genome with sexual selection: reducing mutation load through selection on males. Evolution, 63, 569–582. 19154364 10.1111/j.1558-5646.2008.00558.x

[evl3292-bib-0080] Wilson Sayres, M.A. , Venditti, C. , Pagel, M. & Makova, K.D. (2011) Do variations in substitution rates and male mutation bias correlate with life‐history traits? A study of 32 mammalian genomes. Evolution, 65, 2800–2815. 21967423 10.1111/j.1558-5646.2011.01337.x

[evl3292-bib-0081] Wilton, A.N. & Sved, J.A. (1979) X‐chromosomal heterosis in *Drosophila melanogaster* . Genet. Res, 34, 303–315. 120828 10.1017/s0016672300019534

[evl3292-bib-0082] Xirocostas, Z.A. , Everingham, S.E. & Moles, A.T. 2020 . The sex with the reduced sex chromosome dies earlier: a comparison across the tree of life. Biol. Lett, 16:20190867. 32126186 10.1098/rsbl.2019.0867 PMC7115182

[evl3292-bib-0083] Yun, L. , Chen, P.J. , Kwok, K.E. , Angell, C.S. , Rundle, H.D. & Agrawal, A.F. (2018) Competition for mates and the improvement of nonsexual fitness. Proc. Natl. Acad. Sci. USA, 115, 6762–6767. 29891650 10.1073/pnas.1805435115 PMC6042133

[evl3292-bib-0084] Ziehm, M. , Ivanov, D.K. , Bhat, A. , Partridge, L. & Thornton, J.M. (2015) SurvCurv database and online survival analysis platform update. Bioinformatics, 31, 3878–3880. 26249811 10.1093/bioinformatics/btv463 PMC4653391

