# Supplementary material for: How much does the unguarded X contribute to sex differences in life span?
Source: Evol Lett. 2022 Jul 5;6(4):319–29. doi: 10.1002/evl3.292 (PMC9346086; doi:10.1002/evl3.292)
Supplement: Supplementary file 1 — Supplementary Information [file EVL3-6-319-s002.docx]

**SUPPLEMENTARY MATERIAL**

**Variance for dominance and the unguarded X effect**

Mutation accumulation data suggest that $\bar{h}\approx0.25$, with most dominance coefficients being nonzero and partially recessive (0 < *h_i_* < 0.5; see Manna et al. 2011; Charlesworth 2015), in which case var(*h*) should be small enough to be neglected in our results. For example, if we let *h_i_* follow a beta distribution with shape parameters *a* and *b*, then the mean and variance for *h_i_* is:

$$\bar{h}=\frac{a}{a+b}$$

$$\mathrm{var} \left( h \right)=\frac{ab}{\left( a+b \right)^{2}\left( 1+a+b \right)}=\frac{\bar{h}\left( 1-\bar{h} \right)}{1+a+b}$$

This distribution is compatible with the data when $\bar{h}=0.25$ and $a+b>10$, the latter condition ensuring that most of the distribution is within 0 < *h_i_* < 0.5, and only a small proportion of mutations have dominant or completely recessive fitness effects (*h_i_* > 0.5 and *h_i_* = 0, respectively). In this case, we have $\mathrm{var} \left( h \right)<\frac{3}{176}\approx0.017$, which contributes very little to the unguarded X effect.

**Possible effects of dosage compensation on the unguarded X**

Suppose, following Charlesworth et al. (1987), that a lack of dosage compensation causes a 2-fold reduction in the effect of each X-linked mutation on survival, longevity and fitness of the heterogametic sex relative to the case where there is complete dosage compensation. In our original baseline model with full dosage compensation, we substituted *s_f,i_* = *s_m,i_*  and *α_f,i_* = *α_m,i_* into eq. (4) to obtain:

$$\begin{aligned} \frac{\bar{W}_{f,X}}{\bar{W}_{m,X}}\approx\exp\left( \sum_{i=1}^{n_{X}} \left( 2\mu_{f,i}+\mu_{m,i} \right)\alpha_{i}\frac{1-2h_{i}}{1+2h_{i}} \right)\#\left( S1 \right) \end{aligned}.$$

For the analogous case of a species with no dosage compensation (and, hence, *s_f,i_* = 2*s_m,i_* under the assumptions stated above, and all else remaining the same as the original baseline model), we have:

$$\begin{aligned} \frac{\bar{W}_{f,X}}{\bar{W}_{m,X}}\approx\exp\left( \sum_{i=1}^{n_{X}} \left( 2\mu_{f,i}+\mu_{m,i} \right)\alpha_{i}\frac{1-4h_{i}}{1+4h_{i}} \right)\#\left( S2 \right). \end{aligned}$$

Equations (S1) and (S2) suggest that the basic conditions of dominance that give rise to unguarded X effects differ between the two scenarios of dosage compensation. With complete dosage compensation, the heterogametic sex tends to live longer than the homogametic sex when terms in the summation of eq. (S1) are positive, which requires deleterious mutations tend to be at least partially recessive (*i.e.*, the summation is guaranteed to be positive, and ${\bar{W}_{f,X}}/{\bar{W}_{m,X}}>1$, when $0\leq h_{i}<1/2$). In the absence of dosage compensation, terms in the summation of eq. (S2) are positive under a much more restrictive condition: $0\leq h_{i}<1/4$.

Overall, unguarded X effects are equally strong between the cases of complete dosage compensation and no dosage compensation when deleterious mutations are completely recessive (*h_i_* = 0), in which case eqs. (S1) and (S2) simplify to:

$$\frac{\bar{W}_{f,X}}{\bar{W}_{m,X}}\approx\exp\left( 3U_{X}\bar{\alpha} \right)$$

Otherwise, unguarded X effects are either dampened by incomplete dosage compensation (*e.g*., when dominance falls within the range $0<h_{i}<1/4$), or the effect will be reversed, with the homogametic sex dying earlier than the heterogametic sex when dominance exceeds the threshold $h_{i}=1/4$. These general predictions are illustrated in Figure S1, which compares eqs. (S1) and (S2) under an idealized, though informative, scenario in which dominance coefficients are constant across loci (*h* = *h_i_*).


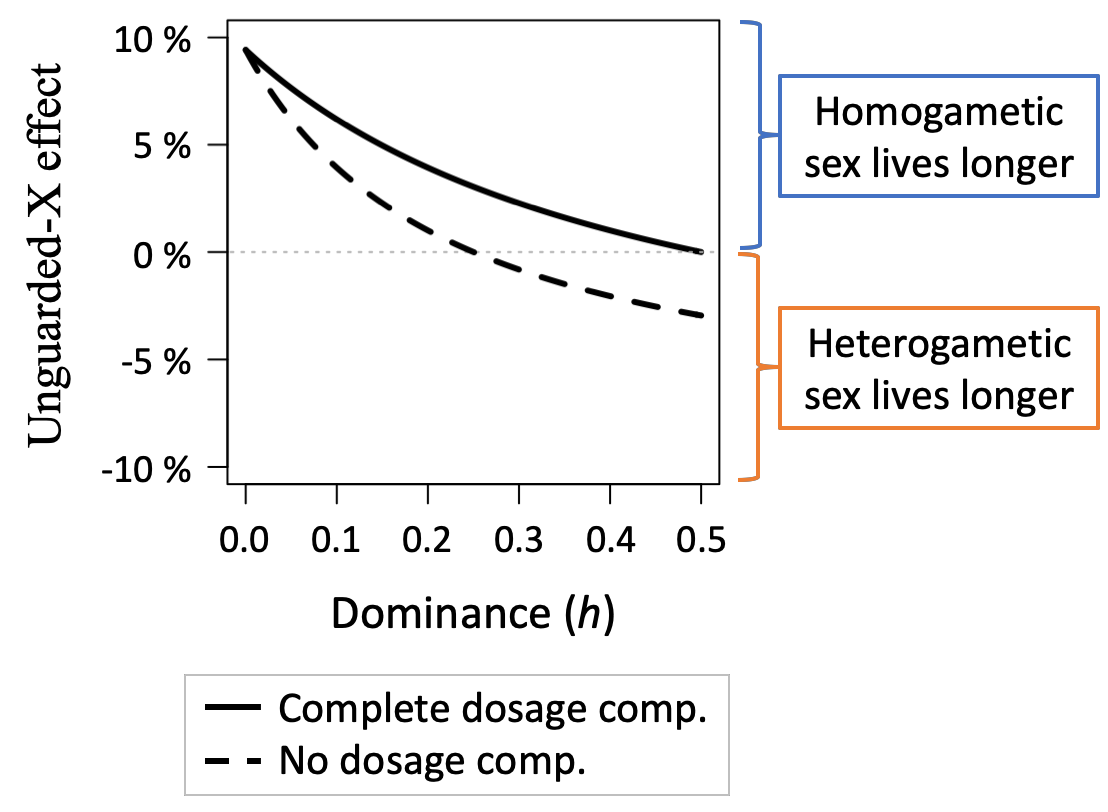


**Figure S1.** Possible effects of dosage compensation on the unguarded-X effect. The curves are based on eqs. (S1) and (S2), with dominance constant across loci (*h* = *h_i_*), and the remaining parameters based on *Drosophila* data (*U_X_* = 0.1 and $\bar{\alpha}$ = 0.3; see the main text for details).

**Lifespan sexual dimorphism in laboratory *Drosophila melanogaster***

We obtained estimates of female and male lifespan from laboratory populations of *Drosophila melanogaster* in the SurvCurv database (Ziehm et al. 2015; <https://www.ebi.ac.uk/thornton-srv/databases/SurvCurv/>). We retained studies that measured lifespan of both sexes, using the same strain and treatment conditions, and those that had large sample sizes (*i.e.*, $n_{H}\geq100$, where $n_{H}$ is the harmonic mean of female and male sample sizes for a given strain and treatment). The dataset, following filtering, included 29 paired estimates (female/male) from 6 studies (see the references, below). Across the 29 estimates, the median sex difference in lifespan was 11.1% longer in females. The average sex difference was 12.6% longer in females, with the 95% CI between 8.7% and 16.5% (CI were calculated using a one-sample t-test in R for the set of estimates of the % sex difference). Estimates and references are presented below.

| Study Reference | Strain & treatment | Harmonic mean sample size | % change in lifespan |
| --- | --- | --- | --- |
| [1] | wDah (Wol+) +200microM rapamycin | 100.0 | 12.3 |
| [1] | wDah (Wol+) 0microM rapamycin | 100.0 | 13.0 |
| [2] | FMR1 WT (w1118) female | 114.4 | 22.4 |
| [3] | w[1118] (dilp7[211] control) females | 100.5 | 20.9 |
| [3] | w[DahT], dilp7[211] homo females | 102.0 | 4.0 |
| [3] | w[1118], dilp7[211] homo females | 128.6 | 8.3 |
| [3] | control females | 196.5 | 11.5 |
| [3] | dilp2[88orange/88orange] females | 199.0 | 10.3 |
| [3] | dilp2[88orange/+] females | 201.0 | 11.1 |
| [3] | dilp2[88orange/213] females | 202.5 | 10.3 |
| [4] | Fly females 25C 1SY | 430.3 | 19.6 |
| [5] | w1118 Lnk[Del29]/Lnk[Del29] females | 160.8 | 5.9 |
| [5] | wDah Lnk[Del29]/+ females | 172.4 | 26.4 |
| [5] | wDah females | 173.4 | 22.6 |
| [5] | wDah Lnk[Del29]/Lnk[Del29] females | 174.4 | 11.3 |
| [5] | Lnk[d07478]/Lnk[d07478] females | 175.5 | 3.1 |
| [5] | w1118 females | 181.6 | 4.9 |
| [5] | w1118 Lnk[Del29]/+ females | 193.8 | 8.5 |
| [5] | w1118 females | 197.0 | 0.0 |
| [5] | Lnk[d07478]/+ females | 199.4 | -3.4 |
| [6] | wDah +/+ female | 100.0 | 8.0 |
| [6] | wDah Indy206/+ 10bc female | 100.0 | 12.5 |
| [6] | wDah Indy206/Indy206 10bc female | 100.0 | 12.5 |
| [6] | wDah Indy206/+ 6bc | 129.6 | 9.1 |
| [6] | wDah +/+ | 139.0 | 9.4 |
| [6] | CS +/+ female | 200.0 | 13.6 |
| [6] | CS 1085/+ female | 200.0 | 52.1 |
| [6] | CS Indy206/+ female | 200.0 | 16.4 |
| [6] | CS Indy302/+ female | 200.0 | 8.6 |

**References**

1. Bjedov I, Toivonen JM, Kerr F, Slack C, Jacobson J, Foley A, Partridge L. 2010. Mechanisms of life span extension by rapamycin in the fruit fly *Drosophila melanogaster*. *Cell Metab* 11(1):35-46.
2. Bushey D, Tononi G, Cirelli C. 2009. The Drosophila fragile X mental retardation gene regulates sleep need. *J Neurosci* 29(7):1948-61.
3. Grönke S, Clarke DF, Broughton S, Andrews TD, Partridge L. 2010. Molecular evolution and functional characterization of Drosophila insulin-like peptides. *PLoS Genet* 6(2):e1000857.
4. Iqbal A, Piper M, Faragher RGA, Naughton DP, Partridge L, Ostler EL. 2009. Chemical changes in aging *Drosophila melanogaster*. *Age* 31(4):343-51.
5. Slack C, Werz C, Wieser D, Alic N, Foley A, Stocker H, Withers DJ, Thornton JM, Hafen E, Partridge L. 2010. Regulation of lifespan, metabolism, and stress responses by the Drosophila SH2B protein, Lnk. *PLoS Genet* 6(3):e1000881.
6. Toivonen JM, Walker GA, Martinez-Diaz P, Bjedov I, Driege Y, Jacobs HT, Gems D, Partridge L. 2007. No influence of Indy on lifespan in Drosophila after correction for genetic and cytoplasmic background effects. *PLoS Genet* 3(6):e95.
